# Computed tomography imaging phenotypes of hepatoblastoma identified from radiomics signatures are associated with the efficacy of neoadjuvant chemotherapy

**DOI:** 10.1007/s00247-023-05793-5

**Published:** 2023-11-20

**Authors:** Yingqian Chen, Matthias F. Froelich, Hishan Tharmaseelan, Hong Jiang, Yuanqi Wang, Haitao Li, Mingyao Tao, Ying Gao, Jifei Wang, Juncheng Liu, Stefan O. Schoenberg, Shiting Feng, Meike Weis

**Affiliations:** 1 Department of Radiology, First Affiliated Hospital, Sun Yat-Sen University, No. 58 Zhongshan Er Lu, Guangzhou, 510080 China; 2 Department of Radiology and Nuclear Medicine, University Medical Center Mannheim, Medical Faculty Mannheim of the University of Heidelberg, Theodor-Kutzer-Ufer 1-3, 68167 Mannheim, Germany; 3 Department of Pediatric Surgery, First Affiliated Hospital, Sun Yat-Sen University, Guangzhou, China

**Keywords:** Computed tomography, Hepatoblastoma, Machine learning, Neoadjuvant chemotherapy, Pediatric

## Abstract

**Background:**

Though neoadjuvant chemotherapy has been widely used in the treatment of hepatoblastoma, there still lacks an effective way to predict its effect.

**Objective:**

To characterize hepatoblastoma based on radiomics image features and identify radiomics-based lesion phenotypes by unsupervised machine learning, intended to build a classifier to predict the response to neoadjuvant chemotherapy.

**Materials and methods:**

In this retrospective study, we segmented the arterial phase images of 137 cases of pediatric hepatoblastoma and extracted the radiomics features using PyRadiomics. Then unsupervised k-means clustering was applied to cluster the tumors, whose result was verified by t-distributed stochastic neighbor embedding (t-SNE). The least absolute shrinkage and selection operator (LASSO) regression was used for feature selection, and the clusters were visually analyzed by radiologists. The correlations between the clusters, clinical and pathological parameters, and qualitative radiological features were analyzed.

**Results:**

Hepatoblastoma was clustered into three phenotypes (homogenous type, heterogenous type, and nodulated type) based on radiomics features. The clustering results had a high correlation with response to neoadjuvant chemotherapy (*P*=0.02). The epithelial ratio and cystic components in radiological features were also associated with the clusters (*P*=0.029 and 0.008, respectively).

**Conclusions:**

This radiomics-based cluster system may have the potential to facilitate the precise treatment of hepatoblastoma. In addition, this study further demonstrated the feasibility of using unsupervised machine learning in a disease without a proper imaging classification system.

**Graphical abstract:**

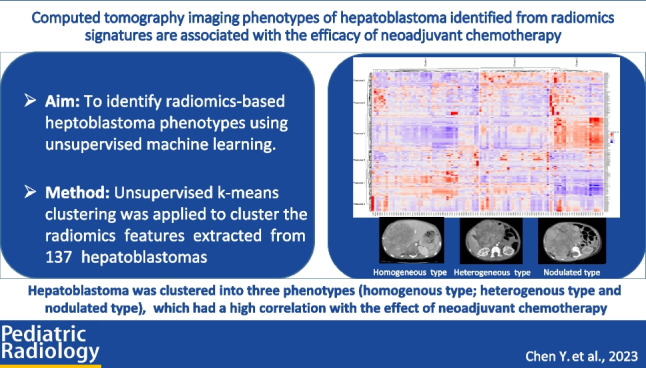

**Supplementary Information:**

The online version contains supplementary material available at 10.1007/s00247-023-05793-5.

## Introduction

Hepatoblastoma, is an embryonal tumor which accounts for two-thirds of malignant liver tumors in children, and makes up between 1% and 2% of all childhood malignancies [1]. The overall survival rate has increased (largely in the past three decades), from approximately 30% to 70%, due to advances in chemotherapy, image modalities, and surgical techniques [2]. But some challenges still prevent it from having a better prognosis.

Most studies have shown that if the tumor is completely resectable, the prognosis is favorable [3]. Up to 70% of patients could not receive surgery directly when the tumors were found. Moreover, tumor recurrence is more often seen in patients without chemotherapy [4]. Thus, neoadjuvant chemotherapy is recommended for most patients, which is critical in increasing the complete tumor resection rate and reducing the recurrence rate [5]. The main therapeutic goal of neoadjuvant chemotherapy is to reduce tumor volume and reach complete surgical resection. Yet, treatment results for advanced tumors are still far from being satisfying. A total of 20% of patients have anticancer drug resistance [6]. For these patients, neoadjuvant chemotherapy not only prolongs the course of treatment but also reduces the degree of tolerance for surgery. Thus, predicting the sensitivity to neoadjuvant chemotherapy is essential for choosing the proper therapeutic schedule for these patients.

However, it is hard to predict the effect of neoadjuvant chemotherapy. Generally, the pathology of the initial diagnosis is most likely to predict the efficacy of chemotherapy. That said, for most cases of hepatoblastoma, the purpose of tumor biopsy is simply to confirm the diagnosis. In clinical practice, although biopsy is recommended by the guidelines (e.g., International Childhood Liver Tumors Strategy Group (SIOPEL) guideline and Expert Consensus for Multidisciplinary Management of Hepatoblastoma (CCCG-HB-2016) protocol), some investigators consider that it may not be necessary for young children (6 months to 3 years of age) with a very high alpha-fetoprotein (AFP) level [7, 8]. Furthermore, the heavily vascularized tumors make it difficult to perform a needle biopsy. In addition being a heterogenous tumor, hepatoblastoma has different compounds and various biologic behaviors [9]; a needle biopsy may not represent the heterogeneity of the tumor.

Compared with the pre-surgery biopsy, the appearance in imaging can provide a comprehensive evaluation of the tumor, not only in regard to location and vascular invasion but also regarding the composition of the tumor. Even now, there is still not a satisfactory imaging classification system, primarily due to the complexity of the appearance in imaging [10]. An objective and quantifiable method is needed to carry out the assessment and clustering of hepatoblastomas with imaging-based intralesional heterogeneity.

Nowadays, with the help of machine learning, massive data analysis is no longer a problem. Radiomics, research focused on the extraction of quantitative metrics, is a rapidly evolving field. Besides esimple lesion characteristics such as shape and heterogeneity, radiomics can extract information out of complex gray value patterns which are difficult to recognize or quantify by human eyes [11]. This characteristic makes radiomics data mineable, which means that it can be used to discover previously unknown patterns of disease evolution, progression, and treatment response with a sufficient dataset. A previous study found that unsupervised clustering may have the potential to classify the image phenotypes by radiomics features [12].

Regarding the clinical challenge, this study aimed to identify radiomics-based hepatoblastoma phenotypes using unsupervised machine learning. The correlations between clusters and clinical and pathological features were analyzed, intending to establish an imaging-based categorization for hepatoblastoma.

## Methods

### Patient enrollment and imaging protocols

In this single-center retrospective study, all patients were enrolled from the First Affiliated Hospital, Sun Yat-sen University and examined between August 2013 and September 2021. This study was approved by the institutional review board of the First Affiliated Hospital of Sun Yat-sen University (No. [2021]027). The inclusion criteria were as follows: (1) initially diagnosed hepatoblastoma with clear pathologic diagnosis; (2) age between 0 and 6 years old; (3) received neoadjuvant chemotherapy based on the CCCG-HB-2016 protocol [13]; and (4) had contrast-enhanced computed tomography (CT) imaging before and after neoadjuvant chemotherapy within 1 week. The exclusion criteria were as follows: (1) incomplete clinical data before and after neoadjuvant chemotherapy (mainly the AFP level); (2) patients receiving interventional therapy during neoadjuvant chemotherapy; and (3) poor quality of contrast-enhanced CT images (evaluated by two experienced radiologists).

All subjects were scanned in our institution, using either a Siemens SOMATOM FORCE®, Siemens Healthcare GmbH (Erlangen, Germany) or a IQon Spectral CT, Philips Healthcare (Amsterdam, Netherlands) scanner. Scanning parameters were assigned based on patient weight, with the use of size-based protocols. Each scan was manually checked for liver lesions and de-identified for further image processing.

### Clinical staging

The stages of the tumor were evaluated using the PRETEXT system of the SIOPEL [14]. The second imaging examination was carried out following two cycles of cytostatic treatment to monitor the effect. The Response Evaluation Criteria in Solid Tumors (RECIST) was used to evaluate the effect of chemotherapy [15].

### Image quality evaluation

**Fig. 1:**
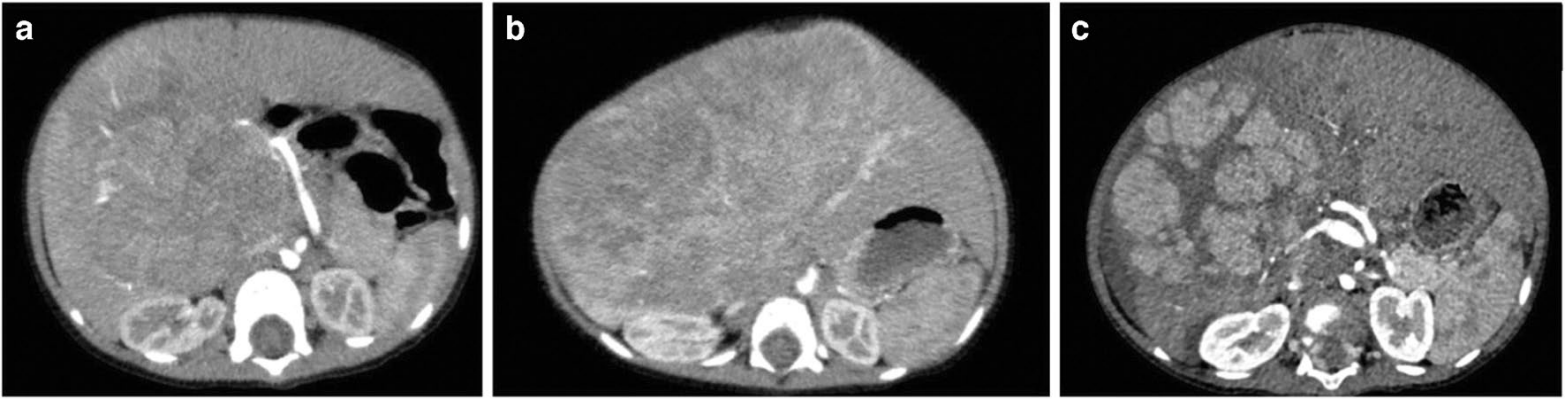
Axial post-contrast computed tomography images in three children with hepatoblastoma show criteria and examples of arterial phase evaluation. **a** Early arterial phase in a 1.5-year-old boy: there is no contrast in the portal vein. **b** Arterial phase in a 1-year-old girl: there is mild enhancement of the portal vein. **c** Late arterial phase in a 1.5-year-old boy: there is clear enhancement of the portal vein (possibly also the vena cava)

Only arterial phase contrast-enhanced axial CT datasets before neoadjuvant chemotherapy were included, because the arterial phase shows the clearest margin and highlights the heterogeneity within the tumor [16]. Image quality was assessed and divided into three categories, excellent, good, and normal quality, in order to retain the comparability of the radiomics properties between individual patients (assessed by Y.C., a clinical radiologist with 7 years of clinical experience in pediatric radiology). However, although we scanned the arterial phase at a similar time in all patients, the enhancement degree was hard to control to an exact phase, due to different circulatory clearance rates in children. To control the effect of the degree of arterial phase enhancement on image quality, the arterial phase was further classified into early arterial phase, arterial phase, and late arterial phase for comparison. The criteria and example images of arterial phase evaluation are shown in Fig. 1 and Supplementary Material 1.

### Radiomics features extraction

All the contast-enhanced CT images were resliced to the resolution ratio of 0.5 mm × 0.5 mm × 1 mm (width × length × height). Following this, segmentations of the tumors were completed manually by a medical student (H.L., three years of experience in pediatric radiology and segmentation), and were then evaluated and corrected by a clinical radiologist (Y.C.) to achieve precise segmentation. If the patient had more than one lesion, only the largest lesion was extracted for analysis.

The free Python software PyRadiomics was used to extract the tumors’ radiomics features (version 3.0.1, Harvard, Boston, MA).The features “firstorder,” “shape,” “glcm” [Gray Level Co-occurrence Matrix], “gldm” [Gray Level Dependence Matrix], “glrlm” [Gray Level Run Length Matrix], “glszm” [Gray Level Size Zone Matrix], and “ngtdm” [Neighboring Gray Tone Difference Matrix] were extracted from the original images as well as from the images after wavelet-transformation (eight wavelet decompositions), resulting in a total of 872 features. Supplemental Material 2 contains a complete list of the extraction settings and parameters used in this analysis. Then the features were exported and prepared for further analysis.

### Unsupervised clustering and feature selection

The statistical analysis was performed in R and RStudio (version 1.3.1093, Boston, MA). Before analysis, each feature was normalized using the Z-score. Then, a line of within-cluster sums of squares was drawn to determine the number of clusters (Supplementary Material 3). A hundred times repeated k-means clustering of tumors and radiomic characteristics were used to separate potentially clinically important cohorts and displayed in an extra heatmap, which also included the hierarchical approach, to cluster the tumors in an unsupervised manner. The outcome of clustering visually was further validated using the t-distributed stochastic neighbor embedding (t-SNE) algorithm, one of the unsupervised approaches for descending dimension.

The least absolute shrinkage and selection operator (LASSO) regression algorithm was used to exclude the redundant features and identify the most relevant features for the differentiation between cluster groups using the “glmnet” package in R [17]. The reduced heatmaps were created for the final feature set. The heatmaps were all created using the “ComplexHeatmap” package in R [18].

### Cluster analysis

Based on the essential features chosen, the unsupervised clustering results were examined quantitatively and visually. Then, the demographic data, image quality, and contract phase between clusters were analyzed. Using a Chi-squared test, it was determined whether there was any link between clinical parameters, pathologic parameters, and qualitative radiological features and the previously identified clusters. SPSS statistical software (version 21.0, IBM Corp., Armonk, NY) was used for statistical analysis, and the measurement data were expressed as x ± s. The Chi-square test was used for the comparison of all categorical variables and the *t*-test was used for dimensional data. A *P*-value of below 0.05 was considered statistically significant.

## Results

### Patient enrollment

**Fig. 2:**
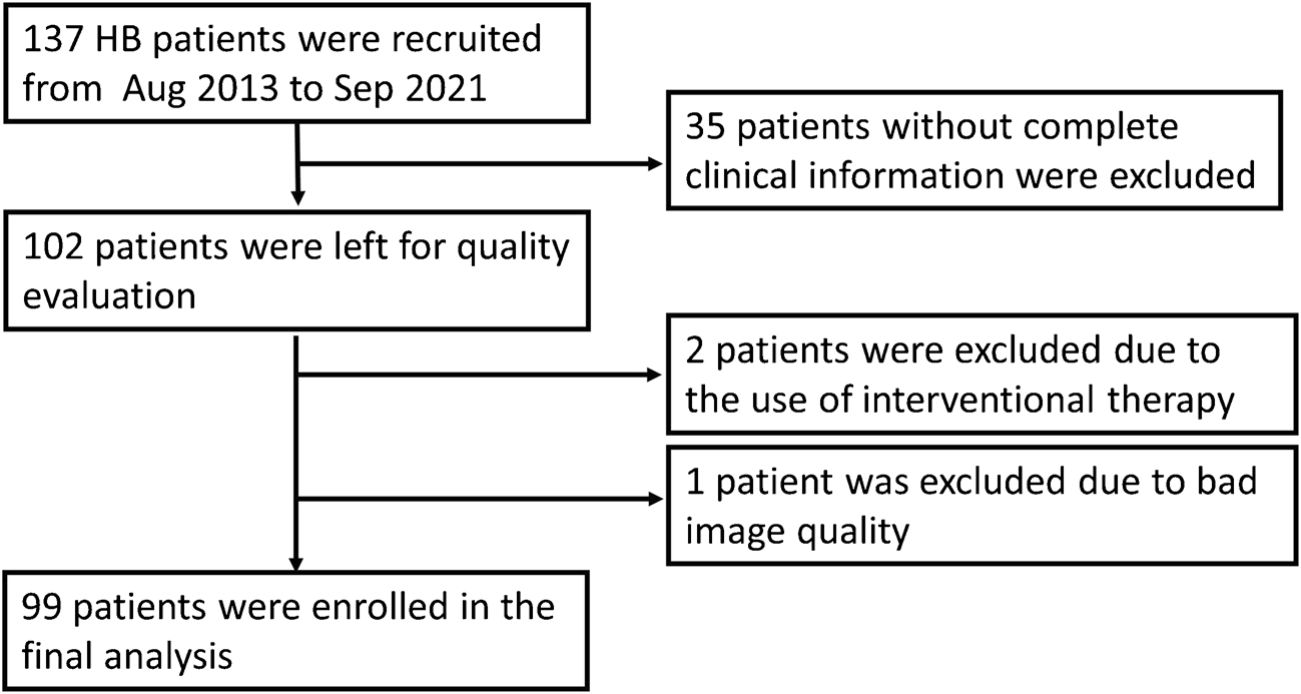
Consort flow chart showing patient recruitment *HB* hepatoblastoma

Based on the inclusion criteria, the contrast-enhanced CT images of 137 patients were enrolled in this study. From these cases, 35 were excluded due to incomplete clinical data, two were excluded due to the use of interventional therapy, and one was excluded because of poor image quality. As a result, 99 patients (54 males), including 99 tumors, were finally enrolled for analysis (Fig. 2). In this cohort, the average age was 30 months (range from 0–163 months). The PRETEXT stages of these patients are shown in Table 2.

### Cluster analysis

**Fig. 3:**
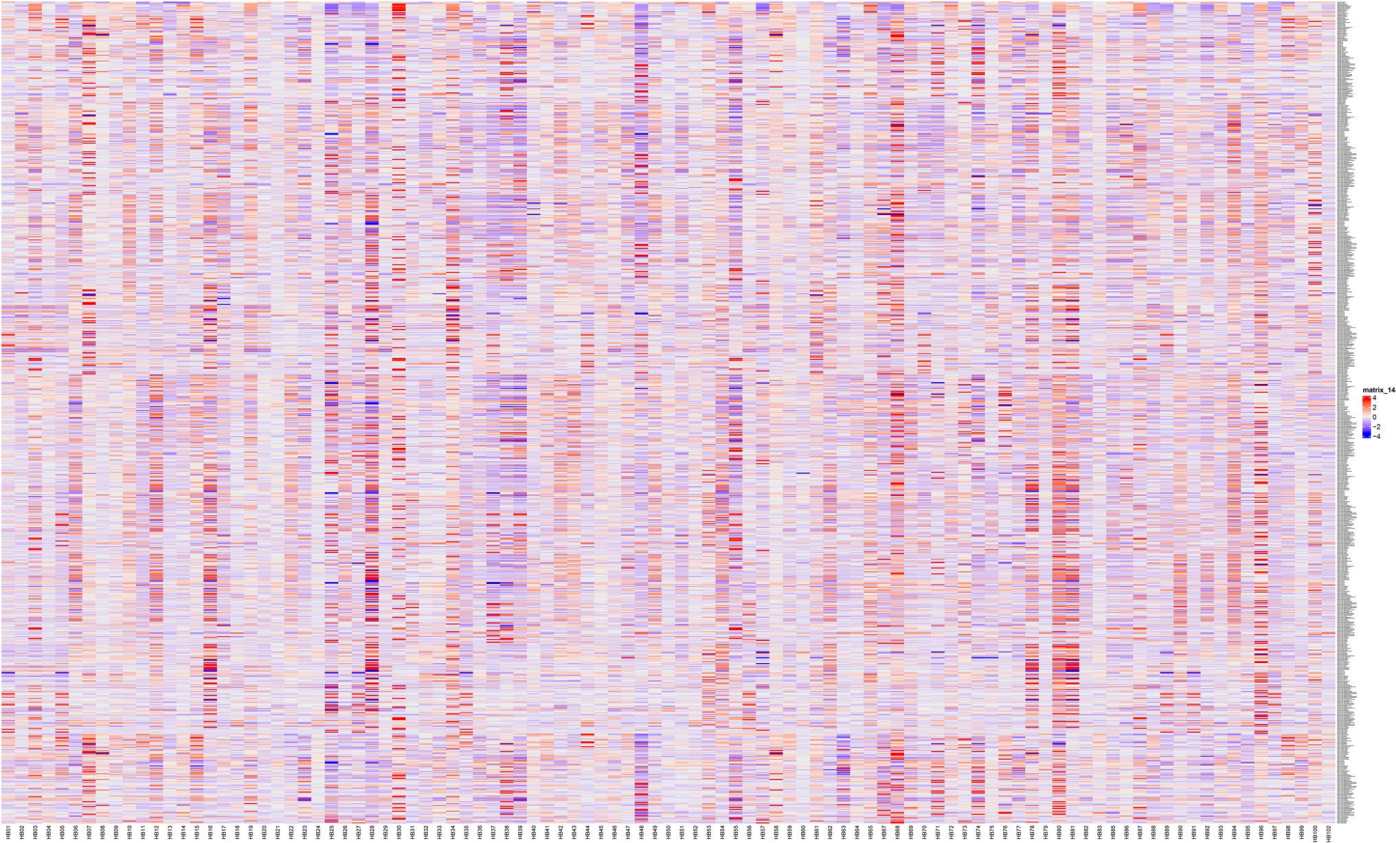
Heatmap of unclustered features shows all extracted features from all patients

**Fig. 4:**
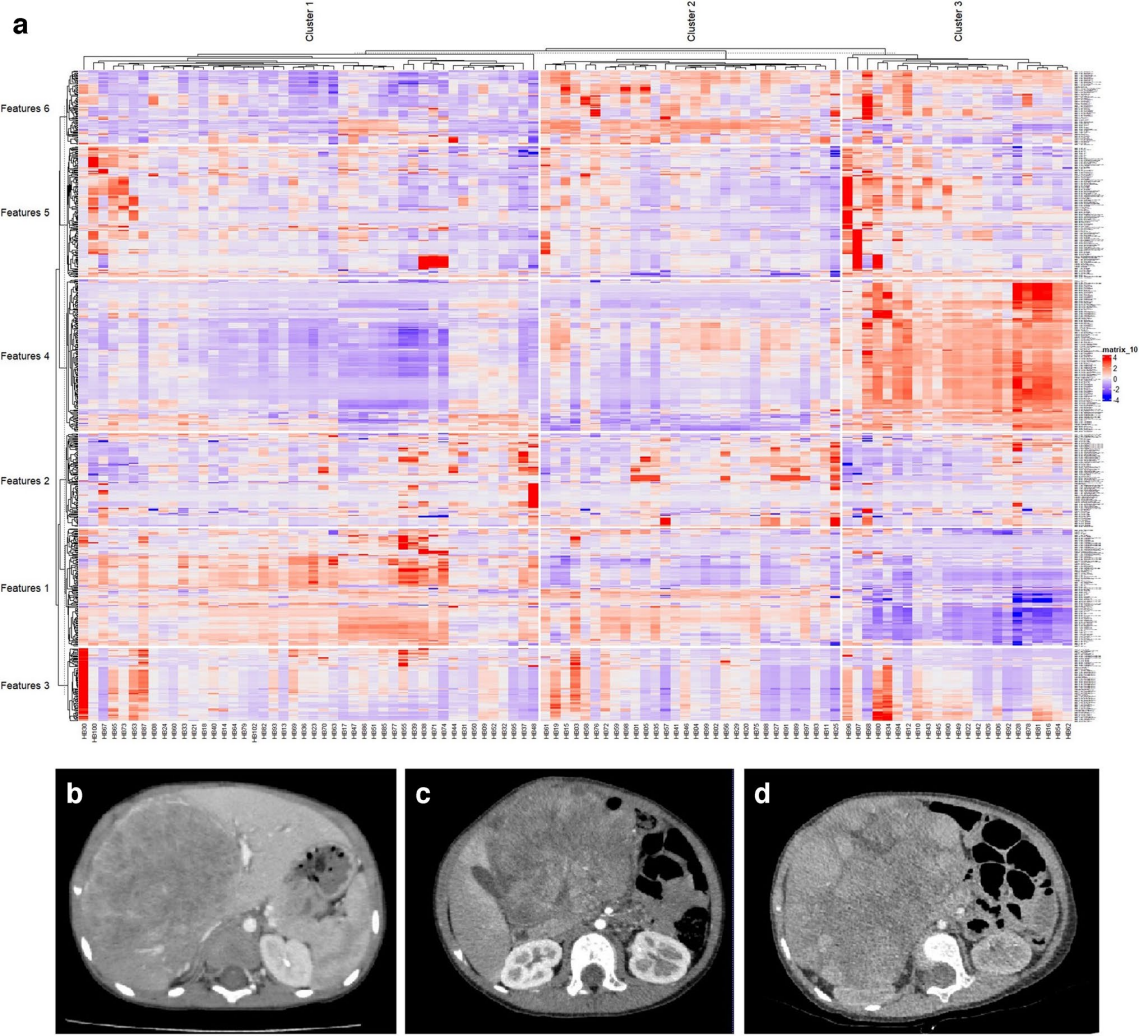
Heatmap of the clustering result and typical images for each cluster. The features were clustered into six groups and the tumors were clustered into three groups (**a**). Hepatoblastoma was clustered into three phenotypes, including homogenous type (**b**), heterogenous type (**c**), and nodulated type (**d**), based on radiomics features

**Fig. 5:**
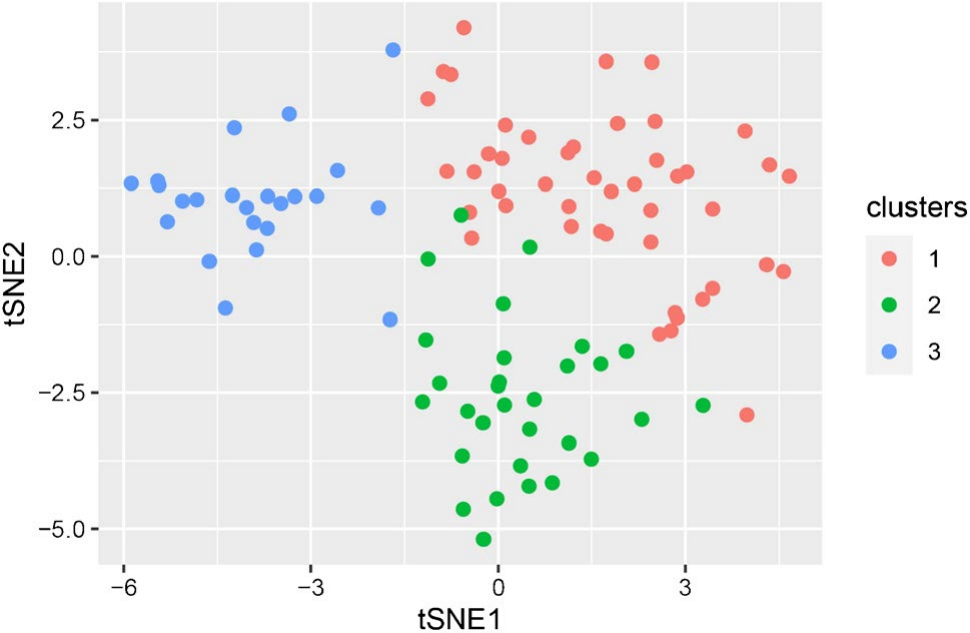
T-distributed stochastic neighbor embedding (t-SNE) result after adding the color of clustering results. *tSNE* t-distributed stochastic neighbour embedding

The k-means clustering method clusters the tumors unsupervised into three groups and the features into six groups. The heatmap of unclustered features and tumors is shown in Fig. 3. The heatmap in Fig. 4 shows the clustering result. The total number of lesions was 46, 30, and 23 for each cluster. The unsupervised t-SNE method further verified the reliability of the classification (Fig. 5).

### Demographic data and imaging quality between clusters

Age, sex, and image phase were comparable in each cluster. However, the image quality showed a statistical difference between clusters (Table 1).

**Table 1:** Demographic data and image quality

Variable		Cluster 1	Cluster 2	Cluster 3	*P*
*n* (cases)		46	30	23	
Age (months) (median, interquartile range)		17 (22)	16 (26)	23 (126)	0.778
Sex	Male	24 (52.2%)	18 (60.0%)	12 (52.2%)	0.772
	Female	22 (47.8%)	12(40.0%)	11(47.8%)	
Image quality	Excellent	30 (65.2%)	20 (66.7%)	10 (43.5%)	**0.030**
	Good	15 (32.6%)	9 (30.0%)	8 (34.8%)	
	Normal	1 (2.2%)	1 (3.3%)	5 (21.7%)	
Image phase	Early arterial phase	7 (15.2%)	3 (10.0%)	4 (9.3%)	0.464
	Arterial phase	26 (56.5%)	23 (38.3%)	14 (32.6%)	
	Late arterial phase	13 (28.2%)	4 (13.3%)	5 (21.8%)	

Bold represents statistical significance (*P*<0.05)

We further analyzed the correlation between image quality and age. The result showed that age was positively associated with image quality (the older the child, the better the image quality, *r*=0.262, *P*=0.009), which meant that although age did not show a statistical difference between the clusters, it did have an effect on image quality.

### Selection of important features

**Fig. 6:**
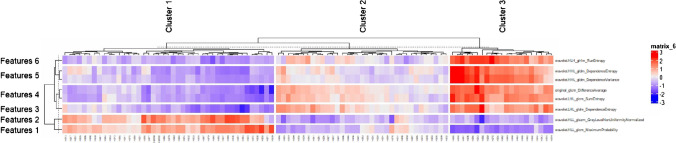
Feature reduced heatmap clustered by lesions, identifying the features “original_glcm_DifferenceAverage” “wavelet.LHL_glcm_SumEntropy” “wavelet.LHL_gldm_DependenceEntropy” “wavelet.HLL_glcm_MaximumProbability” “wavelet.HLL_glszm_GrayLevelNonUniformityNormalized” “wavelet.HLH_glrlm_RunEntropy” “wavelet.HHL_gldm_DependenceEntropy” and “wavelet.HHL_gldm_DependenceVariance”. *glcm*: gray-level co-occurrence matrix; *gldm*: gray level dependence matrix; *glrlm*: gray-level run-length matrix; *glszm*: gray-level size zone matrix;  *HHL*: high-pass high-pass low-pass; *HLH*: high-pass low-pass high-pass; *HLL*: high-pass low-pass low-pass; *LHL*: low-pass high-pass low-pass

By using the supervised lasso regression method with the dependent variable of clusters, the most relevant eight features were selected (Supplementary Material 4), including “original_glcm_DifferenceAverage,” “wavelet.LHL_glcm_SumEntropy,” “wavelet.LHL_gldm_DependenceEntropy,” “wavelet.HLL_glcm_MaximumProbability,” “wavelet.HLL_glszm_GrayLevelNonUniformityNormalized,” “wavelet.HLH_glrlm_RunEntropy,” “wavelet.HHL_gldm_DependenceEntropy,” and “wavelet.HHL_gldm_DependenceVariance.” The features are shown as a heatmap in Fig. 6.

### Visual cluster analysis

Two experienced radiologists (Y.C and M.F., a clinical radiologist with seven years’ experience) visually analyzed the hepatoblastoma within the clusters. Thus, the categories were assigned according to their imaging characteristics as (i) homogenous type, (ii) heterogenous type, and (iii) nodulated type. The typical images are shown in Fig. 4.

### Differences in clinical parameters, pathological parameters, and qualitative radiological features among different clusters

The correlations of clinical parameters and pathologic parameters with the three previously defined clusters are shown in Table 2. The PRETEXT stages showed no difference between clusters. However, the effect of neoadjuvant chemotherapy, assessed by RECIST, did show a significant difference between clusters. Among these clusters, the homogenous type tended to have the best therapeutic effect, while the nodulated type tended to resist chemotherapy. When comparing the pathological epithelial ratio of the clusters (only 74 cases were assessed in pathologic testing), the homogenous type and heterogenous type had higher epithelial components than the nodulated type.

**Table 2:** Clusters and patient clinical and pathological characteristics

Variable		Cluster 1	Cluster 2	Cluster 3	*P*
*n* (cases)		46	30	23	
PRETEXT	Stage 1	2 (4.3%)	3 (10.0%)	1 (4.3%)	0.274
	Stage 2	11 (23.9%)	14 (46.7%)	8 (34.8%)	
	Stage 3	26 (56.5%)	12 (40.0%)	12 (52.2%)	
	Stage 4	7 (15.2%)	1 (3.3%)	2 (8.7)	
RECIST (adjuvant chemotherapy)	PR	28 (60.9%)	16 (53.3%)	8 (34.8%)	**0.02**
	SD	18 (39.1%)	13 (43.3%)	11 (47.8%)	
	PD	0 (0.0%)	1 (3.3%)	4 (17.4%)	
AFP (× 10^5^ μg/L)		5.35 ± 4.45	5.54 ± 6.38	4.02 ± 5.59	0.612
Epithelial ratio(*n*=74)		89.7 ± 18.0 (*n*=35)	90.1 ± 19.4 (*n*=23)	81.6 ± 27.3 (*n*=16)	**0.029**

Bold represents statistical significance (*P*<0.05)

*PD*, progressive disease; *PR*, partial response; *SD*, stable disease

When comparing the qualitative radiological features between the clusters, most of the common image signs, including the capsule state, margin state, intratumoral hemorrhage, and calcification, did not show the difference between the groups. Only the cystic change inside the tumor showed between-cluster differences (Table 3). Besides, we also assessed the other parameters of the tumor, including growing pattern (inside or outside of the liver), tumor location (left lobe or right lobe), contiguous extrahepatic tumor, multifocality, tumor rupture, caudate lobe involvement, lymph node metastasis, and distant metastasis, which also did not show between-cluster difference (Supplementary Material 5).

**Table 3:** Clusters and patient qualitative radiological features

Variable		Cluster 1	Cluster 2	Cluster 3	*P*
*n* (cases)		46	30	23	
Capsule	No capsule	7 (15.2%)	6 (20.0%)	2 (8.7%)	0.613
	Incomplete capsule	11 (23.9%)	8 (26.7%)	9 (39.1%)	
	Complete capsule	28 (60.9%)	16 (53.3%)	12 (52.2%)	
Margin	Smooth	12 (26.1%)	10 (33.3%)	6 (26.1%)	0.811
	Lobulated	18 (39.1%)	12 (40.0%)	40 (40.4%)	
	Irregular	4 (8.7%)	2 (6.7%)	0	
	Invasive	12 (26.1%)	6 (20.0%)	7 (30.4%)	
Intratumoral Hemorrhage	With	38 (82.6%)	28 (93.3%)	19 (82.6%)	0.371
	Without	8 (17.4%)	2 (6.7%)	4 (17.4%)	
Calcification	With	23 (50.0%)	20 (66.7%)	12 (52.2%)	0.336
	Without	23 (50.0%)	10 (33.3%)	11 (47.8%)	
Cystic change	With	16 (34.8%)	20 (66.7%)	15 (65.2%)	**0.008** (**0.04** after FDR-corrected)
	Without	30 (65.2%)	10 (33.3%)	8 (34.8%)	

Bold represents statistical significance (*P*<0.05)

## Discussion

This study for the first time subdivides hepatoblastoma into three phenotypes based on radiomics features, using unsupervised clustering. The clustering results demonstrated good predictability of the impact of neoadjuvant chemotherapy and the pathogenic component, demonstrating the clinical utility of this method. This radiomics-based clustering system may have the potential to facilitate the precise treatment of hepatoblastoma. Additionally, this study showed that unsupervised algorithms can be successfully applied to diseases like hepatoblastoma that lack a reliable imaging classification method.

The reliability of unsupervised clustering is the basis of this study. There are four kinds of mainstream unsupervised clustering algorithms, including the k-means cluster method, hierarchical clustering, t-SNE cluster method, and density-based spatial clustering of applications with noise (DBSCAN) [19]. Theoretically, each algorithm has its randomness. To avoid the resulting bias, we repeated the k-means clustering algorithm 100 times to obtain relatively stable results. Additionally, the “ComplexHeatmap” package in R, which was used to draw the heatmap in our study, embeds the hierarchical method. Thus, the heatmap result also showed the result of clustering by hierarchical method, which was comparative with the result by the k-means method. Additionally, we also used the t-SNE approach for validation, further demonstrating the validity and dependability of the clustering result.

Previous studies have also tried to predict the histopathology and the prognosis of hepatoblastoma using contrast-enhanced CT characteristics in a conventional way. But the performance was shown to be poor [20]. It is simple to discover by evaluating the quantitative radiological features between the clusters that the traditional radiological characteristics have little impact on the clustering outcome, highlighting the benefit of radiomics analysis. Roughly, radiomics features include histogram-based and texture-based, model-based, transform-based, and shape-based features [21]. The more extensive and varied radiomics properties allow for the discovery of previously unidentified markers. In this way, one can use a similar method to explore the new image-based clustering system, as well as its important features.

Among the eight important features selected by LASSO, one came from the unfiltered original group, and the other seven came from the wavelet transform filtered group. Three of them were Gray-Level Co-occurrence Matrix (GLCM) features. The GLCM, as a second-order gray-level histogram, describes spatial relationships of pairs of voxels or voxels with predefined gray-level intensities [11]. A further three features were calculated from the Gray Level Dependence Matrix (GLDM), which quantifies gray level dependencies in an image [22]. The final two features came from Gray Level Size Zone Matrix (GLSZM) and Gray Level Run-length Matrix (GLRLM). In short, these features all represent the gray-level variation from distinct perspectives. This suggests that, in contrast to the shape, diameter, and other histogram data, the tumoral texture is the crucial point to differentiate hepatoblastoma.

Developed from degenerated hepatoblasts, hepatoblastoma can be differentiated according to the liver development stages. The tumor can be subdivided into two main histologic subtypes: epithelial type and mixed type [23]. Generally, in tumors, the epithelial component is more sensitive to chemotherapy than the mesenchymal component [24]. In hepatoblastoma, similarly, compared with mixed type, epithelial type has a better prognosis [25]. As we previously discussed, the intensity variance is the primary source of variation between the clusters. And usually, the intensity variance inside the tumor indicates the component variance of the tumor. Additionally, it has been demonstrated that tumors with poor response to neoadjuvant chemotherapy typically have a poor prognosis [4]. What’s more, the cystic change inside the tumor can reflect the heterogeneity to a certain extent. These could help explain why, according to our findings, tumors in cluster 3 had the greatest degree of image heterogeneity, the worst response to neoadjuvant treatment, and the lowest epithelial ratio in histology.

Finally, we saw that the most widely used classification scheme—PRETEXT—does not exhibit a high link with the clusters in this study. As we know, the PRETEXT system, which describes the extent of the tumor within the liver, focuses on the resectability of the tumor [26]. This imaging clustering approach can be utilized in conjunction with the PRETEXT system since it highlights the susceptibility to neoadjuvant chemotherapy.

This study is a preliminary single-center study. Since a ground truth for training cannot be easily defined, the unsupervised approach has its intrinsic limitations. Though we tried different algorithms to improve the credibility of this work, more detailed pathologic data and more external data from other institutions is needed to verify the clinical utility of this approach. Recent studies have reported some genetic phenotypes and epigenetic footprint of hepatoblastoma, which might correlate with chemotherapy resistance and prognosis [27, 28]. Regrettably, in this retrospective study, we were not able to test the genetic phenotype of these tumors. Further study is needed to identify a possible correlation between the clustering system and gene expression in hepatoblastoma. Lastly, the predictive ability of the clustering system to determine long-term prognosis was not assessed and should be another goal for futture studies.

## Conclusion

In conclusion, we grouped hepatoblastoma into three clusters based on radiomics features. This new classification system may help to predict the response to neoadjuvant chemotherapy , potentially promoting precision medical care for patients with hepatoblastoma. 

### Supplementary Information

Below is the link to the electronic supplementary material.

Supplementary file1 (DOCX 142 KB)

## Data Availability

Data will be made available on reasonable request.
