# Supplementary material for: Computed tomography imaging phenotypes of hepatoblastoma identified from radiomics signatures are associated with the efficacy of neoadjuvant chemotherapy
Source: Pediatr Radiol. 2023 Nov 20;54(1):58–67. doi: 10.1007/s00247-023-05793-5 (PMC10776468; doi:10.1007/s00247-023-05793-5)
Supplement: Supplementary file 1 — Supplementary file1 (DOCX 142 KB) [file 247_2023_5793_MOESM1_ESM.docx]

**Supplementary material 1:** The criteria and example images of image quality evaluation

Excellent quality: excellent image quality with high signal-noise ratio

Good quality: image with normal signal-noise ratio

Normal quality: image with a small amount of beam hardening artifacts

**
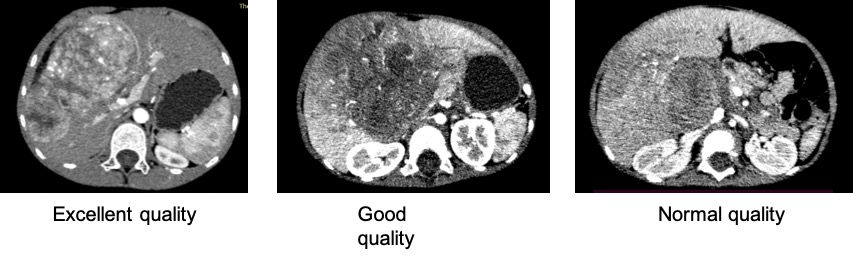
**

**Supplementary material 2:** Pyradiomics Settings Extraction parameters:

{'minimumROIDimensions': 2, 'minimumROISize': None, 'normalize': False, 'normalizeScale': 1, 'removeOutliers': None, 'resampledPixelSpacing': None, 'interpolator': 'sitkBSpline', 'preCrop': False, 'padDistance': 5, 'distances': [1], 'force2D': False, 'force2Ddimension': 0, 'resegmentRange': None, 'label': 1, 'additionalInfo': True}
Enabled filters:

{'Original': {}; ‘Wavelet’: {}}

Enabled features:

{'firstorder': [], 'glcm': [], 'gldm': [], 'glrlm': [], 'glszm': [], 'ngtdm': [], 'shape': []}

**Supplementary material 3:** A line of within-cluster sums of squares for determination of the cluster number


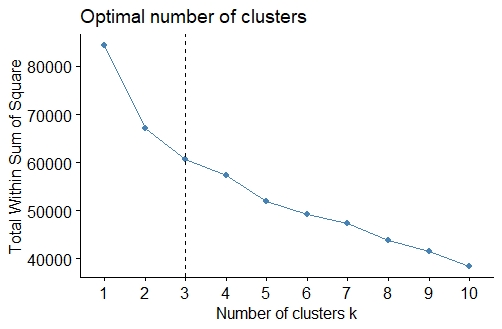


**Supplementary material 4:** Results of LASSO regression

| Feature | Regression coefficient |
| --- | --- |
| original_glcm_DifferenceAverage | 0.492010371 |
| wavelet.LHL_glcm_SumEntropy | 0.690191141 |
| wavelet.LHL_gldm_DependenceEntropy | 0.015796334 |
| wavelet.HLL_glcm_MaximumProbability | -0.169760644 |
| wavelet.HLL_glszm_GrayLevelNonUniformityNormalized | -0.649323666 |
| wavelet.HLH_glrlm_RunEntropy | 2.570781560 |
| wavelet.HHL_gldm_DependenceEntropy | 0.597154936 |
| wavelet.HHL_gldm_DependenceVariance | 0.001922463 |

**Supplementary material 5:**

| Variable |  | Cluster 1 | Cluster 2 | Cluster 3 | p |
| --- | --- | --- | --- | --- | --- |
| n (cases) |  | 46 | 30 | 23 |  |
| Growing pattern | Inside the liver | 35 (76.1%) | 19 (63.3%) | 16 (69.6%) | 0.486 |
|  | Outside the liver | 11 (23.9%) | 11 (36.7%) | 7 (30.4%) |  |
| Tumor location | Left lobe | 6 (13.0%) | 5 (16.7%) | 3 (13.0%) | 0.255 |
|  | Right lobe | 6 (13.0%) | 10 (33.3%) | 5 (21.7%) |  |
|  | Both lobes | 34 (73.9%) | 15 (50.0%) | 15 (65.2%) |  |
| Contiguous extrahepatic tumor (E) | Yes | 45 (97.8%) | 28 (93.3%) | 23 (100%) | 0.336 |
|  | No | 1 (2.2%) | 2 (6.7%) | 0 |  |
| Multifocality (F) | 0 | 38 (82.6%) | 26 (87.6%) | 19 (82.6%) | 0.881 |
|  | ＞1 | 8 (17.4%) | 4 (13.3%) | 4 (17.4) |  |
| Tumor rupture (R) | Yes | 4 (8.7%) | 1 (3.3%) | 1 (4.3%) | 0.585 |
|  | No | 42 (91.3%) | 29 (96.7%) | 22 (95.7%) |  |
| Caudate involvement (C) | Yes | 14 (33.3%) | 7 (23.3%) | 9 (39.1%) | 0.463 |
|  | No | 22 (66.7%) | 23 (76.6%) | 14 (60.9%) |  |
| Lymph node metastasis (N) | Yes | 41 (89.1%) | 27 (90.0%) | 22 (95.7%) | 0.660 |
|  | No | 5 (10.9%) | 3 (10.0%) | 1 (4.3%) |  |
| Distant metastasis (M) | Yes | 38 (82.6%) | 29 (96.7%) | 19 (82.6%) | 0.163 |
|  | No | 8 (17.4%) | 1 (3.3%) | 4 (17.4%) |  |
